# Prognostic Value and Potential Immunoregulatory Role of SCARF1 in Hepatocellular Carcinoma

**DOI:** 10.3389/fonc.2020.565950

**Published:** 2020-09-29

**Authors:** Daniel A. Patten, Alex L. Wilkinson, Joanne M. O'Rourke, Shishir Shetty

**Affiliations:** National Institute for Health Research Birmingham Liver Biomedical Research Unit and Centre for Liver and Gastrointestinal Research, Institute of Immunology and Immunotherapy, University of Birmingham, Birmingham, United Kingdom

**Keywords:** scavenger receptor, leukocyte recruitment, tumor endothelial cells, liver cancer, tumor microenviroment

## Abstract

Scavenger receptor class F member 1 (SCARF1) is thought to play an important role in the selective recruitment of CD4^+^ T cells to liver sinusoidal endothelial cells during chronic liver disease. However, the contribution of SCARF1 to hepatocellular carcinoma (HCC) is currently unknown. We utilized publically-available RNA-sequencing data from The Cancer Genome Atlas (TGCA) to explore *SCARF1* expression in HCC and correlated it with a number of clinicopathological features. Flow adhesion assays were used to determine the role of SCARF1 in CD4^+^ T cell subset recruitment. SCARF1 expression was downregulated in HCC tumor tissues, compared to non-tumoral tissues, and loss of *SCARF1* expression was associated with poorly differentiated/aggressive tumors. Additionally, higher *SCARF1* expression in HCC tumor tissues was highly prognostic of better overall, disease-free and progression-free survival. SCARF1 within HCC was largely associated with tumor endothelial cells and adhesion studies suggested that it played a role in the specific recruitment of proinflammatory CD4^+^ T cells (CD4^+^CD25^−^) to HCC tumor tissues. Endothelial SCARF1 expression in tumor biopsies may provide critical prognostic information. Additionally, SCARF1 may also be a novel endothelial target that could help re-programme the microenvironment of HCC by promoting effector T cell tumor infiltration.

## Introduction

Globally, hepatocellular carcinoma (HCC) is the second most common cause of cancer-related deaths and its incidence is predicted to further increase (1). Due to a combination of poor surveillance and lack of conclusive biomarkers (2), a large majority of HCC patients present with advanced disease and, consequently, current interventional therapies can only act to prolong survival by a few months. In more than 90% of cases, HCC occurs on the background of chronic liver disease/cirrhosis and thus provides a paradigm for inflammation-induced cancer (3). It is well-known that tumor-infiltrating lymphocytes (TILs) significantly influence the tumor microenvironment and their phenotype strongly influences prognosis in HCC (4–6); consequently, immunotherapies for the treatment of HCC are receiving increasing attention in the literature (7, 8). Present research is predominantly focussed on the efficacy of checkpoint blockade inhibitors (CIs) to “remove the brake” on the immune system in order to provide an anti-tumoral immune response; however, recent success of CIs with anti–vascular endothelial growth factor (VEGF) treatment has highlighted the importance of the endothelium in the context of immunotherapy (9). Only a subset of patients with HCC appear to respond to immunotherapy, but selecting which patients will benefit continues to be a challenge and the presence or absence of TILs in HCC is likely to play a significant role in the response to immunotherapy. Despite this, the endothelial pathways and molecules involved in the entry of TILs to HCC tumors are considerably understudied. Lymphocyte recruitment to the liver occurs within the specialized low flow channels of the sinusoids and via a sequential step-wise process known as the “leukocyte adhesion cascade” (10). The leukocyte adhesion cascade is mediated by a number of receptor-ligand interactions between the lymphocytes and liver sinusoidal endothelial cells (LSEC) and previous studies from our lab and others have implicated members of the scavenger receptor super-family in the recruitment of leukocytes to LSEC *in vitro* (11–16). We have also shown that these endothelial-expressed scavenger receptors are present within the sinusoids of HCC tumor tissues (13, 14); however, their role in shaping the tumor microenvironment via the recruitment of TILs has not been studied to date.

Scavenger receptors are a large super-family of proteins which are defined by their ability to bind and endocytose a vast range of endogenous and exogenous ligands, eliciting the “scavenging” of unwanted macromolecules from the bloodstream (17). Functionally, scavenger receptors generally play beneficial roles in tissue homeostasis and protective roles during infection, but have also been implicated in the persistence of inflammatory disorders, including chronic liver diseases (17, 18) and cancers (19). Liver sinusoidal endothelial cells (LSEC) express an array of scavenger receptors at high density, a phenotype which is consistent with their primary biological function of removing gut-derived antigens from the portal blood (10). However, we have also reported that LSEC-expressed scavenger receptors perform an important secondary role in which they mediate the recruitment of leukocytes to the liver (11).

Scavenger receptor class F, member 1 (SCARF1 or SR-F1), also known as scavenger receptor expressed by endothelial cells (SREC)-I, was first identified in cDNA libraries from human umbilical vein endothelial cells (HUVEC) (20). SCARF1 has been shown to bind and internalize modified low density lipoproteins (LDLs), specifically acLDLs (21), and a wide range of other endogenous damage-associated products (22), such as heat-shock proteins (HSPs) (23–25) and apoptotic host cells (26, 27). In addition to a diverse range of endogenous ligands, SCARF1 also binds a wide array of viral (28–30), fungal (31), and bacterial (32–35) antigens. Furthermore, our lab was the first to comprehensively characterize SCARF1 expression in human liver tissues and primary LSEC and we were able to demonstrate that SCARF1 plays a role in the selective recruitment of CD4^+^ T cells to the sinusoidal endothelium under physiological shear stress (14). In this regard, we hypothesized that SCARF1 actively contributed to the hepatic microenvironment and played an important role in the pathophysiology of chronic inflammatory liver diseases and malignancies (14).

Here, through the utilization of the publically-available TGCA (The Cancer Genome Atlas) RNA-sequencing datasets (http://cancergenome.nih.gov), we describe the differential regulation of scavenger receptors in HCC tumor tissues, compared to non-tumorous control tissues, and specifically focussed on the downregulation of *SCARF1* expression. We corroborated these findings with immunohistochemical staining, which also showed reduced protein expression in HCC tumor tissues, and next explored the relationship of *SCARF1* expression with tumor progression. Consequently, we found an association with loss of *SCARF1* expression with aggressive tumor biology. Following this, we evaluated the prognostic value of *SCARF1* expression in HCC tumors by generating survival curve data, via KM Plotter (http://kmplot.com/analysis/). In support of the pathological findings, high *SCARF1* expression in HCC tumor tissues was found to correlate with a better overall survival, disease-free survival and progression-free survival. Next, via a combination of TGCA data analysis and immunofluorescent staining, we determined that SCARF1 within HCC was largely associated with tumor endothelial cells. Finally, we extended our previous findings with primary human liver endothelial cells by studying subsets of CD4^+^ T cells. Using flow-based adhesion assays under physiological levels of shear stress our findings suggested that SCARF1 could play a role in the recruitment of proinflammatory CD4^+^ T cells (CD4^+^CD25^−^), rather than immunosuppressive T cell subsets, to the HCC tissue microenvironment. Our results demonstrate that SCARF1 could be a prognostic biomarker in HCC. Furthermore, SCARF1 expression could potentially be targeted to alter the inflammatory status of the tumor microenvironment, shifting it toward an anti-tumoral immune response and supporting immunotherapy regimes for HCC.

## Materials and Methods

### *In silico* Data Analysis

Publically-available data from the The Cancer Genome Atlas (TGCA) was utilized throughout this study. To explore scavenger receptor family expression in tumor and relevant non-tumorous tissue controls from the TGCA dataset, the University of California Santa Cruz (UCSC) Xena tool (https://xenabrowser.net/) was used. Correlation of SCARF1 expression with tumor progression/aggression and cell-specific markers was performed via the cBioPortal website (https://www.cbioportal.org/) (accessed 25th Feb 2020). With the use of the publically-accessible tool KM Plotter (http://kmplot.com/analysis/), survival data was generated from the TGCA dataset over a 60-month time period, with the data being split into two groups (“High” and “Low”) by the median of SCARF1 expression. Resultant data was exported to Prism® 6 software (GraphPad Software Inc.) and survival curves were produced. The Gene Expression Profiling Interactive Analysis (GEPIA) website (http://gepia.cancer-pku.cn/) was used to generate a list of the top 25 genes regulated in conjunction with SCARF1 in HCC tumor tissues. Level of CD4^+^ T cell infiltration of HCC tumors was correlated with SCARF1 expression via the Tumor IMmune Estimation Resource (TIMER; https://cistrome.shinyapps.io/timer/; accessed 12th May 2020).

### Human Tissue Samples

All liver tissue samples were collected from patients undergoing transplantation for chronic liver disease or primary hepatocellular carcinoma (HCC) at the University Hospitals Birmingham NHS Foundation Trust, with written informed consent and local ethics committee approval. All experiments were performed in accordance with the regulations and guidelines sanctioned by the West Midlands—South Birmingham Research Ethics Committee, Birmingham, UK (LREC reference 06/Q2702/61 and 04/Q2708/41).

### Immunohistochemistry

Immunohistochemical staining was performed on 7 μm thick acetone-fixed cryosections, stored at −20°C. Prior to staining, sections were thawed to room temperature (RT) and hydrated with PBS/0.1% Tween® 20 (PBST) for 5 min. Endogenous peroxidase activity was then blocked with 0.3% hydrogen peroxide in methanol and blocking of non-specific binding was performed by incubation with 2X Casein Solution (Vector Laboratories, Inc.). Sections were incubated with anti-SCARF-1 primary antibody (8 μg/ml; Abcam; ab92308) diluted in PBS for 1 h at RT and then washed twice in PBST for 5 min. Isotype matched controls at appropriate concentrations were performed in all experiments. Subsequently, sections were incubated with the anti-rabbit ImmPRESS™ HRP for 30 min at RT. Excess secondary antibody was washed off with PBST for 5 min (twice) and sections were then incubated with DAB chromogen (Vector Laboratories Inc.) for 2 min; the reaction was stopped with the addition of distilled H_2_O. Nuclei were then counterstained with Mayer's Hematoxylin (Pioneer Research Chemicals Ltd.) for 30 s and slides were washed in warm H_2_O for 2 min. Sections were subsequently dehydrated in sequential washes of alcohol (3×) and xylene (3×) and mounted using DPX (Phthalate-free) mounting medium (CellPath). Images were taken using an Axio ScanZ1 microscope (ZEISS). Surface area coverage of SCARF1 staining was performed by via threshold analysis using ImageJ software. Five random high-power fields of view were analyzed per section, with the average value taken for each matched pair of tumor and non-tumorous tissues.

### Immunofluorescence

For immunofluorescent staining, 7 μm acetone-fixed cryosections were thawed and then blocked for non-specific binding by incubation in PBS with 10% goat serum and casein solution, for 30 min at RT. This was followed by 1 h incubation with primary antibodies for SCARF-1 (8 μg/ml, Abcam ab92308) and CD31 (5 μg/ml, DAKO JC70A). Samples were washed three times in PBS followed by 30 min incubation with Alexa Fluor® conjugated secondary antibodies (1:500 dilution; Thermo Fisher Scientific). Nuclei were stained with 300 nM DAPI (Invitrogen) and slides were subsequently mounted with ProLong™ Gold Antifade Mountant (Invitrogen). Fluorescence images were acquired using a Zeiss 780 Zen confocal fluorescence microscope (ZEISS).

### LSEC Isolation and Culture

Liver sinusoidal endothelial cells (LSEC) were isolated from ~30 g human liver tissue as described previously (36). Briefly, tissues were subjected to enzymatic digestion via collagenase (10 mg/ml collagenase IA; Sigma-Aldrich) and non-parenchymal cells were separated out via density gradient centrifugation on a 33%/77% Percoll (GE Healthcare) gradient at 800 × g for 25 min. The relevant cell layer was then removed, and LSEC were isolated by positive immunomagnetic selection using CD31 antibody-conjugated DynabeadsTM (Invitrogen). LSEC were then seeded in rat tail collagen (1 in 100; Sigma-Aldrich)-coated culture vessels in medium composed of human endothelial serum-free media (SFM; Invitrogen) supplemented with 10% human serum (HD Supplies), 10 ng/ml vascular endothelial growth factor (VEGF; PeproTech), and 10 ng/ml hepatocyte growth factor (HGF; PeproTech). All cells were grown and maintained at 37°C in a humidified incubator with 5% CO_2_.

### Primary Lymphocyte Isolation

Peripheral blood mononuclear cells (PBMCs) were isolated from whole blood via density gradient centrifugation; briefly, whole blood was layered on Lympholyte®-H (Cedarlane) and centrifuged at 800 × g for 25 min. The PBMC layer was removed and washed in PBS with 2% FCS and 1 mM EDTA (Gibco™ by Thermo Fisher Scientific) and centrifuged at 800 × g for 5 min. A platelet depletion step was then performed by a second wash in PBS with 2% FCS and 1 mM EDTA and centrifugation at 350 × g for 10 min. CD4^+^CD25^+^ T lymphocytes were subsequently isolated from PBMCs by Dynabeads™ Regulatory CD4^+^/CD25^+^ T Cell Kit, and in accordance with manufacturer's instructions. The CD4^+^/CD25^−^ fraction obtained via this isolation protocol was kept and used as an “effector” population in flow-based adhesion assays, as previously described (13).

### Flow-Based Adhesion Assays

Flow-based adhesion assays over monolayers of LSEC (13, 37) were used to study lymphocyte recruitment *in vitro*, under conditions of physiological flow. Briefly, approx. 7.5 × 10^5^ LSEC were seeded in rat tail collagen-coated μ-slide VI 0.4 and grown to confluence overnight. Cells were then stimulated with 10 ng/ml TNFα for 24 h to induce endothelial activation. CD4^+^CD25^+^ or CD4^+^CD25^−^ T lymphocytes were isolated (see ‘Primary Lymphocyte Isolation’ above) and resuspended at a cell density of 1 × 10^6^ cells/ml in a flow medium of Endothelial SFM with 0.1% BSA. Lymphocytes were then perfused over the LSEC at a physiological shear of 0.05 Pa, with each channel of the μ-slide perfused for 5 min. Subsequently, channels were washed though for 3 min with flow media alone, after which video recordings were taken. All flow assays were imaged via phase-contrast microscopy on an Olympus IX50 Inverted Microscope (Olympus) and 12 frames from each channel were analyzed. The number of adherent lymphocytes was firstly counted and then normalized to cells/mm^2^/10^6^ cells perfused using the following equation: adherent cells/flow rate (0.28 ml/min) × bolus (5 min) × field of view area (0.154 mm^2^) × (1/ concentration of lymphocytes 1 × 10^6^ cells/ml). The addition of SCARF-1 blocking antibody (10 μg/ml; Abcam; ab92308) or rabbit polyclonal negative control (10 μg/ml; DAKO) was performed immediately preceding each assay and incubated for 30 min (14).

### Statistical Analyses

All data were tested for normal distribution by the D'Agostino-Pearson omnibus test. All data were found to be non-parametric and so were expressed as median ± interquartile range (IQR), with the number of experimental repeats (n) specified in each case. For single comparisons, statistical significance was determined by Mann–Whitney *U*-test, whereas evaluation of multiple treatments was performed by Kruskall–Wallis one-way analysis of variance with *post hoc* Dunn's test. Statistical significance of paired data was calculated via a paired *T*-test. A *p*-value of ≤ 0.05 was considered as statistically significant. All statistical analyses were undertaken using Prism® 6 software (GraphPad Software Inc.).

## Results

### SCARF1 Expression Is Downregulated in HCC Tumors

**Figure 1:**
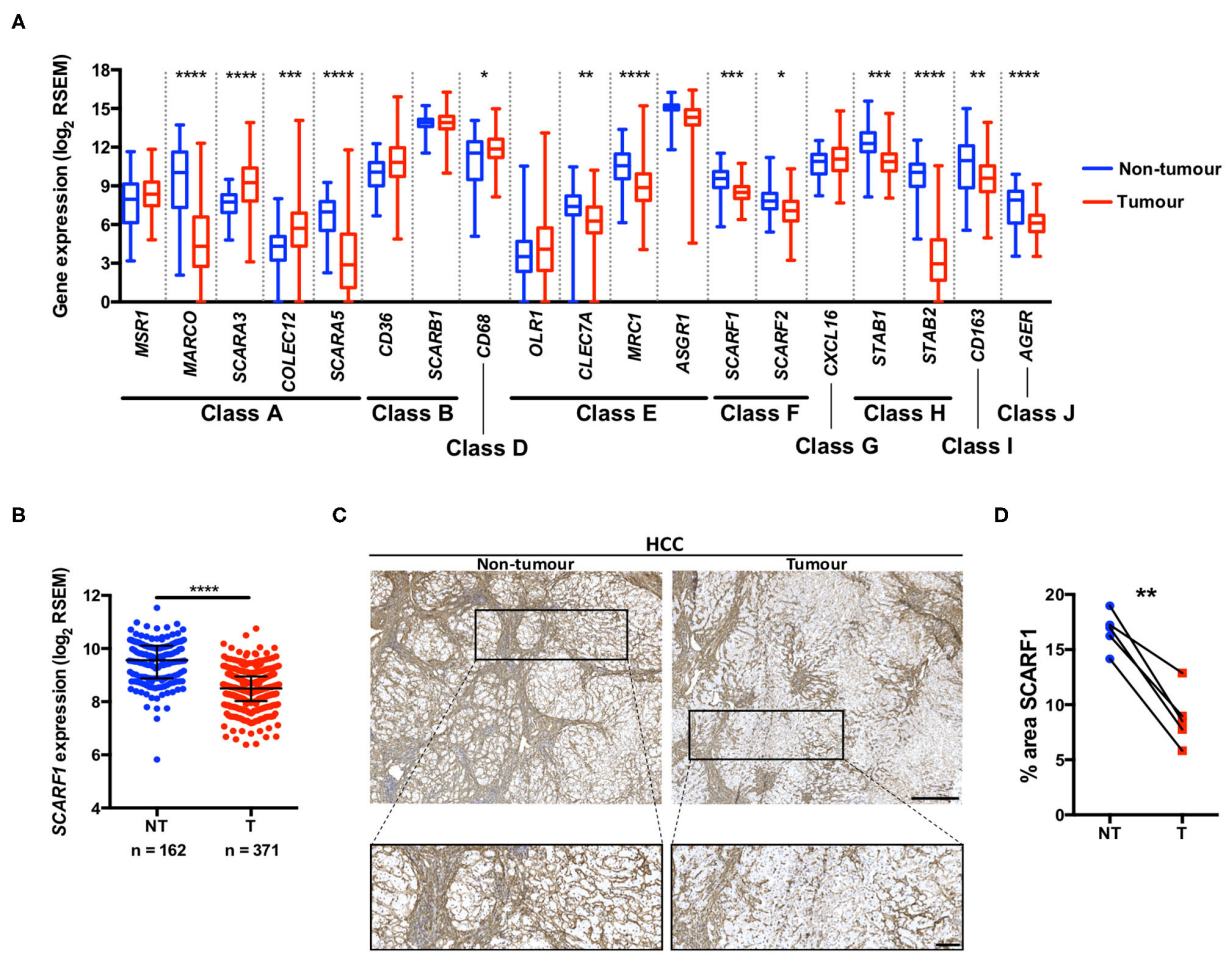
SCARF1 gene and protein expression is downregulated in HCC tumors. **(A)** Regulation of scavenger receptor gene expression in HCC tumors (blue; *n* = 371), compared to non-tumoral tissues (red; *n* = 162). *, **, *** and **** are representative of statistical significance as measured by the Kruskal–Wallis test, where *p* ≤ 0.05, *p* ≤ 0.01, *p* ≤ 0.005, and *p* ≤ 0.001, respectively. RSEM = RNA-Seq by Expectation Maximization. **(B)** Comparison of SCARF1 gene expression in non-tumoral (NT) tissues with HCC tumor tissues (T). ****Indicates statistical significance as measured by Mann–Whitney *U*-test, where *p* ≤ 0.001 **(C)**. Representative images of SCARF1 immunohistochemical staining (brown) in HCC tumor (Right panels) and matched distal, non-tumorous (Left panels) tissues from the same patient. Scale bar = 400 μm; zoomed image scale bar = 100 μm. **(D)** Surface area quantification of immunohistochemical staining in matched HCC tumor (T) and non-tumorous (NT) tissues was performed by via threshold analysis using ImageJ software. **Indicates statistical significance as measured by a paired *T*-test, where *p* ≤ 0.01. *n* = 5, with the average of 5 random high-power fields of view taken per section. Data in **(A,B)** was generated from the TGCA dataset using the University of California Santa Cruz (UCSC) Xena tool (https://xenabrowser.net/).

A number of scavenger receptors have previously been shown to be significantly dysregulated within tumor tissues and have consequently been implicated in the pathophysiology of a wide variety of cancers (19). Here, we explored the mRNA expression of the scavenger receptor super-family in HCC and highlight that a number of members exhibited differential regulation in tumor tissues, compared to non-tumorous control tissues. Interestingly, the majority of scavenger receptors (*MARCO, SCARA5, CLEC7A, MRC1, SCARF1, SCARF2, STAB1, STAB2, CD163, AGER*) demonstrated significantly decreased expression in HCC tumor, compared to non-tumorous liver tissues (Figure 1A). However, in contrast, *SCARA3, COLEC12*, and *CD68* were all up-regulated (Figure 1A) and several others (*MSR1, CD36, OLR1, ASGR1*, and *CXCL16*) did not exhibit any regulation (Figure 1A). Of those significantly regulated, we were particularly interested in those previously implicated in leukocyte recruitment to the liver and specifically focussed on SCARF1 in the current study.

Utilizing qPCR analysis, we have previously shown a strong trend for decreased SCARF1 mRNA expression in HCC tumor tissue compared to normal liver tissue (14); here, we corroborated this finding with publically-available RNA-sequencing data from The Cancer Genome Atlas (TGCA). Analysis of the TGCA data showed that *SCARF1* expression is significantly (*p* ≤ 0.001) lower in HCC tumor tissues in comparison to non-tumorous tissues (Figure 1B). In addition, *SCARF1* expression is also reduced in tumor tissues of other gastrointestinal cancers, namely esophageal carcinoma, stomach adenocarcinoma and colon adenocarcinoma, compared to their respective non-tumorous tissue controls (Figure S1). Interestingly, and in contrast to the other cancer types explored here, pancreatic adenocarcinoma tumors showed no dysregulation of *SCARF1* expression when compared to non-tumorous tissues (Figure S1). Next, we confirmed the downregulation of SCARF1 expression in tumors at the protein level by immunohistochemical staining of HCC tumors and matched distal, non-tumorous tissues (Figure 1C). Surface area quantification of SCARF1 staining in matched samples from several patients showed a significant (*p* ≤ 0.01) reduction in SCARF1 expression in tumor tissues, when compared to distal, non-tumorous tissues (Figure 1D).

### Loss of SCARF1 Expression Is Associated With More Advanced and Aggressive Tumors

**Figure 2:**
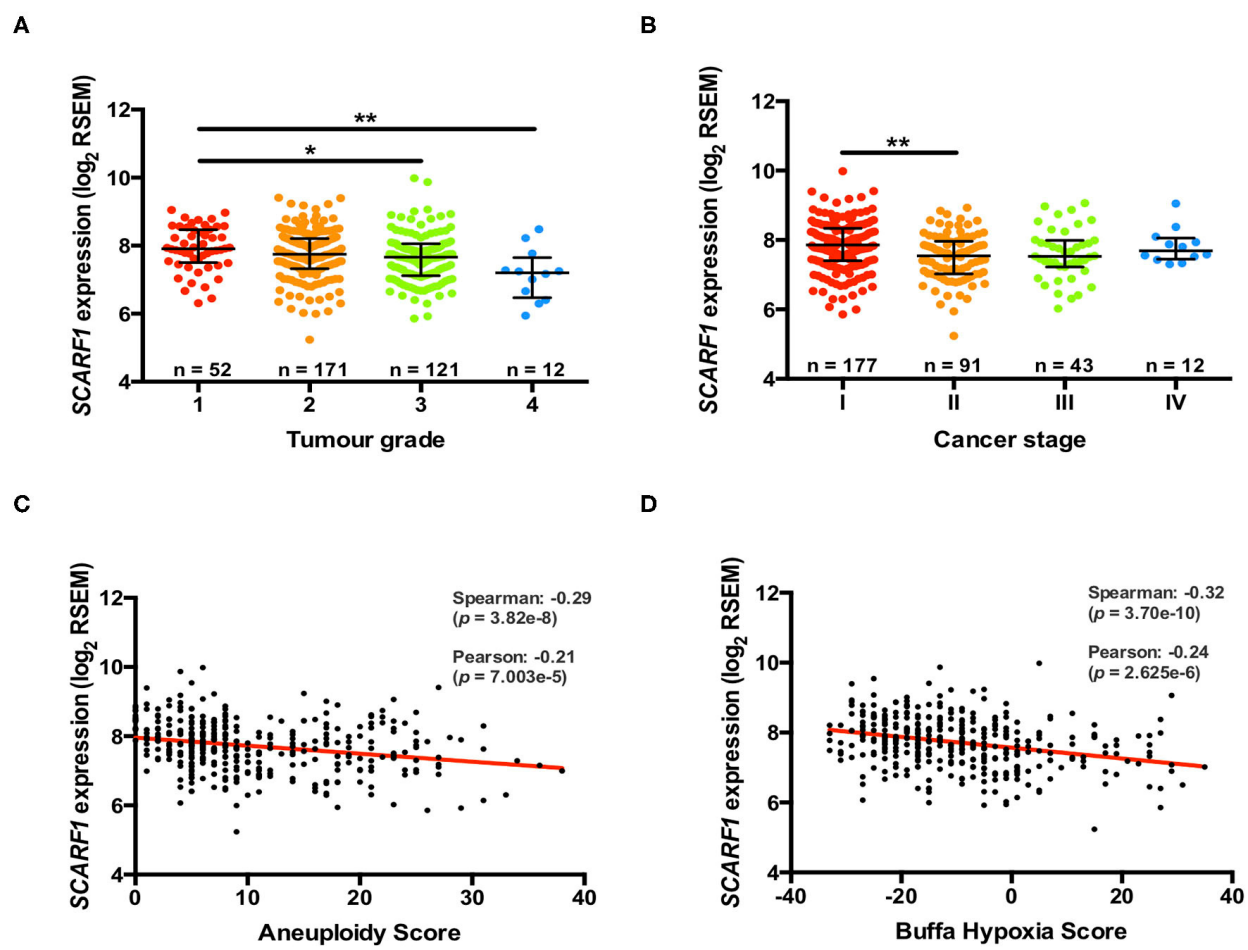
More advanced and aggressive tumors exhibit lower SCARF1 expression. **(A)** SCARF1 expression in HCC tumor tissues of different histological grade. * and ** are representative of statistical significance as measured by the Kruskal–Wallis test, where *p* ≤ 0.05 and *p* ≤ 0.01, respectively. **(B)** SCARF1 expression in HCC tumor tissues from the four cancer stages. **indicates statistical significance as measured by the Kruskal–Wallis test, where *p* ≤ 0.01. SCARF1 expression correlated to tumor aggression parameters **(C)**. Aneuploidy score (*n* =355) and **(D)** Buffa Hypoxia score (*n* = 361). Data in this Figure was generated from the TGCA dataset using the cBioPortal website (https://www.cbioportal.org/) (accessed 25th Feb 2020).

Previously, we have shown that the level of immunohistochemical staining of SCARF1 in poorly differentiated HCC tumor tissues was greatly reduced when compared with well- and moderately-differentiated tumors (14); here, we aimed to utilize the TGCA dataset to further corroborate those findings. Differentiation status of solid tumors informs their histological grading and, consequently, gives an indication of tumor aggressiveness; therefore, we initially explored the expression of *SCARF1* in HCC tumors of different histological grades. In doing this, we showed significantly reduced levels in Grade 3 (*p* ≤ 0.05) and Grade 4 (*p* ≤ 0.01) tumors, when compared to Grade 1 tumors (Figure 2A). Next, we explored the *SCARF1* expression levels in cases of HCC at different stages of the disease, from early stage disease (Stage I) through to highly developed and metastatic disease (Stage IV). When compared to patients with Stage I disease, cohorts of patients with Stages II, III and IV disease all demonstrated a trend for decreased *SCARF1* expression; however, only the data for the Stage II cohort was calculated to be statistically significant (*p* ≤ 0.01) (Figure 2B). We further aimed to confirm these findings by correlating *SCARF1* expression with other parameters commonly associated with increased tumor aggressiveness and grade, in particular, we focussed on Aneuploidy Score (38) and Buffa Hypoxia Score (39). In both instances, *SCARF1* expression demonstrated a moderate negative correlation in HCC tumor tissues (Figures 2C,D), thus providing further evidence that a loss of SCARF1 expression is associated with adverse biology and aggressive HCC tumors.

### Prognostic Value of *SCARF1* Expression in HCC

**Figure 3:**
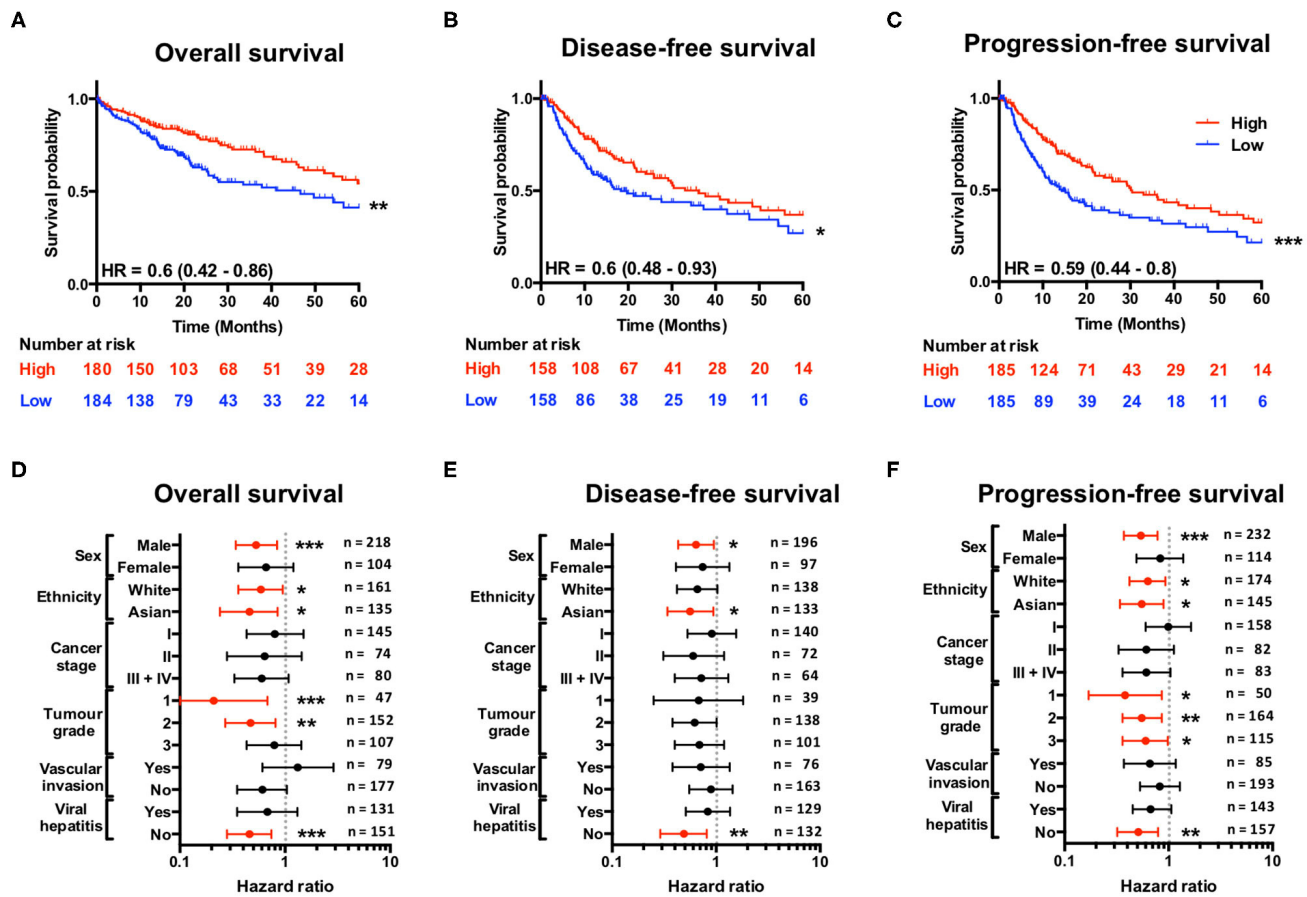
SCARF1 expression is predictive of survival in HCC. **(A)** Overall survival, **(B)** Disease-free survival, and **(C)** Progression-free survival of HCC patients separated into two groups (“High” and “Low” expression) via the median expression of SCARF1. *, ** and *** indicate statistical significance where *p* ≤ 0.05, *p* ≤ 0.01, or *p* ≤ 0.005, respectively. HR = hazard ratio. Forest plots of **(D)** Overall survival, **(E)** Disease-free survival, and **(F)** Progression-free survival in relation to various clinicopathological features of HCC patients. Data is displayed as hazard ratio with 95% confidence intervals. **(D–F)** Red plots highlight clinicopathological parameters in which statistical significance was achieved. *, ** and *** indicate statistical significance where *p* ≤ 0.05, *p* ≤ 0.01 or *p* ≤ 0.005, respectively. Data in this Figure was generated with use of KM Plotter (http://kmplot.com/analysis/).

Having found that a loss of *SCARF1* expression correlates with more advanced and aggressive tumors, we next sought to investigate its prognostic value in HCC. With regards to overall survival, high expression of *SCARF1* was highly indicative of a better prognosis (HR = 0.60, 95% CI = 0.42–0.86, *p* ≤ 0.01; Figure 3A and Figure S2. Interestingly, with the exception of *ASGR1*, which is expressed in HCC tumor cells and known to prevent metastasis (40), *SCARF1* was the only other scavenger receptor gene which was associated with increased survival in HCC (Figure S2). A high expression of *SCARF1* was also associated with a better prognosis when disease-free survival (HR = 0.60, 95% CI = 0.48–0.93, *p* ≤ 0.05; Figure 3B) and progression-free survival (HR = 0.59, 95% CI = 0.44–0.80, *p* ≤ 0.01; Figure 3C) were considered. We also assessed the prognostic value of *SCARF1* expression in correlation with a range of clinicopathological features. In male patients, higher *SCARF1* expression was suggestive of better overall survival (HR = 0.53, 95% CI = 0.34–0.84, *p* ≤ 0.005; Figure 3D), disease-free survival (HR = 0.64, 95% CI = 0.43–0.95, *p* ≤ 0.05; Figure 3E) and progression-free survival (HR = 0.54, 95% CI = 37–0.78, *p* ≤ 0.005; Figure 3F), but, surprisingly, showed no prognostic value in female patients (Figures 3D–F). In Asian patient cohorts, higher *SCARF1* expression was strongly associated with better overall (HR = 0.46, 95% CI = 0.24–0.85, *p* ≤ 0.05; Figure 3D), disease-free (HR = 0.56, 95% CI = 0.34–0.94, *p* ≤ 0.05; Figure 3E) and progression-free (HR = 0.55, 95% CI = 0.34–0.89, *p* ≤ 0.05; Figure 3F) survival; however, in white patients it was indicative of better overall (HR = 0.59, 95% CI = 0.36–0.95, *p* ≤ 0.05; Figure 3D) and progression-free survival (HR = 0.63, 95% CI = 0.42–0.93, *p* ≤ 0.05; Figure 3F), but not disease-free survival (Figure 3E). With regards to histological grade of HCC tumors, a higher *SCARF1* expression was strongly associated with better overall survival (HR = 0.21, 95% CI = 0.07–0.68, p ≤ 0.005; Figure 3D) and progression-free survival (HR = 0.38, 95% CI = 0.17–0.86, *p* ≤ 0.05; Figure 3F) in Grade 1 tumors, but held no prognostic value for disease-free survival (Figure 3E). High expression of *SCARF1* in Grade 2 HCC tumors was again indicative of improved overall survival (HR = 0.47, 95% CI = 0.27–0.81, *p* ≤ 0.05; Figure 3D) and progression-free survival (HR = 0.55, 95% CI = 0.34–0.89, *p* ≤ 0.05; Figure 3F), but showed no effect on disease-free survival (Figure 3E). Higher SCARF1 expression in Grade 3 HCC tumors was only associated with better progression-free survival (HR = 0.60, 95% CI = 0.36–0.98, *p* ≤ 0.05; Figure 3F), but had no prognostic value for overall (Figure 3D) or disease-free survival (Figure 3E). *SCARF1* expression was, however, highly prognostic of better overall survival (HR = 0.46, 95% CI = 0.28–0.74, *p* ≤ 0.005), disease-free survival (HR = 0.49, 95% CI = 0.29–0.81, *p* ≤ 0.01) and progression-free survival (HR = 0.51, 95% CI = 0.32–0.79, *p* ≤ 0.01) in patients with non-viral HCC, but exhibited no prognostic value in viral HCC patients. Furthermore, expression of *SCARF1* showed no prognostic value with regards to cancer staging or in the presence/absence of vascular invasion (Figures 3D–F).

### HCC Tumor-Expressed SCARF1 Exhibits a Strong Endothelial Signature

**Figure 4:**
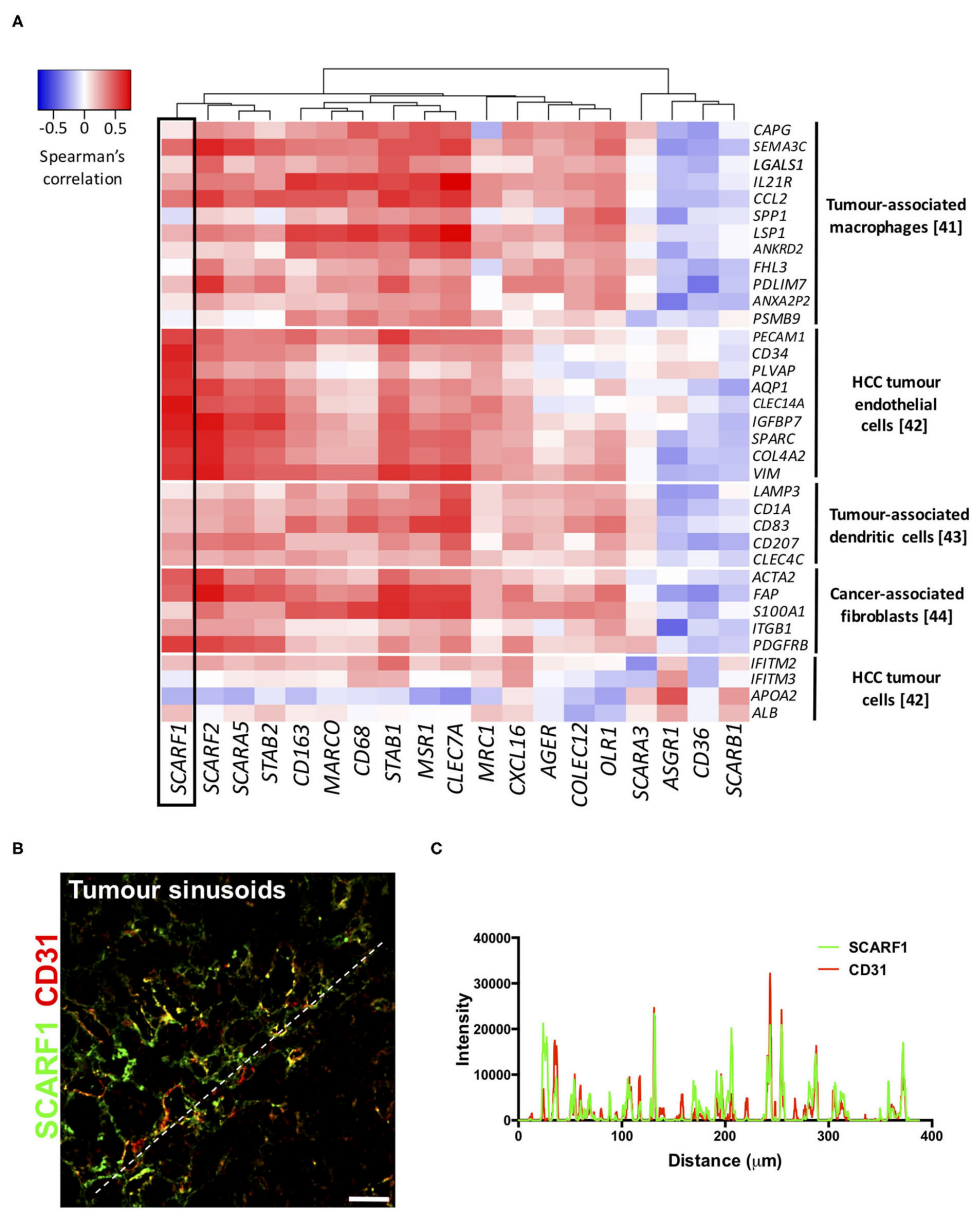
SCARF1 is expressed in HCC tumor endothelial cells. **(A)** Correlation of scavenger receptor gene expression with tumor-associated cell-specific markers. This analysis was performed via the cBioPortal website (https://www.cbioportal.org/) (accessed 10th August 2020). *n* = 358. The heatmap was generated with use of the Heatmapper website (http://www.heatmapper.ca/). The black box highlights the expression profile of *SCARF1*. **(B)** Representative image of dual color immunofluorescent staining of SCARF1 (green) and CD31 (red) within HCC tumor sinusoids. Scale bar = 40 μm. White dashed line delineates site of intensity measurements. **(C)** Intensity measurements of immunofluorescent staining shown in **(B)**.

To explore the cell-specific expression of *SCARF1* within HCC tumors, we correlated the gene expression of the scavenger receptor superfamily with a number of gene sets known to be expressed in tumor-associated cell populations (41–44). Interestingly, of the entire scavenger receptor superfamily, *SCARF1* demonstrated the most endothelial-specific signature within HCC tumor tissues, exhibiting low to moderate correlations with the majority of the other cell type gene sets (Figure 4A). We next utilized the publically-available tool Gene Expression Profiling Interactive Analysis (GEPIA; http://gepia.cancer-pku.cn/) to generate a list of genes commonly regulated in conjunction with *SCARF1* within HCC tumor tissues (Table S1). Out of the top 25 hits, a number of genes were endothelial-specific and we selected some of these to further explore their relationship with *SCARF1* expression. Consequently, all of the endothelial markers selected (*ADGRF5, CD93, FLT4, MMRN2, ESAM, PEAR1, PECAM1, TIE1*, and *CLEC14A*) exhibited a highly significant positive correlation with *SCARF1* expression (Figure S3). Next, to corroborate the expression of *SCARF1* in tumor sinusoidal endothelial cells we undertook dual immunofluorescence staining of SCARF1 and CD31, a commonly used tumor endothelial marker known to be expressed in HCC (42, 45). Within HCC tumor tissue, we demonstrated a strong co-localization of SCARF1 and CD31 (Figures 4B,C).

### SCARF1 Preferentially Supports Adhesion of CD4^+^CD25^−^ “Effector” T Cells to Human Liver Endothelial Cells

**Figure 5:**
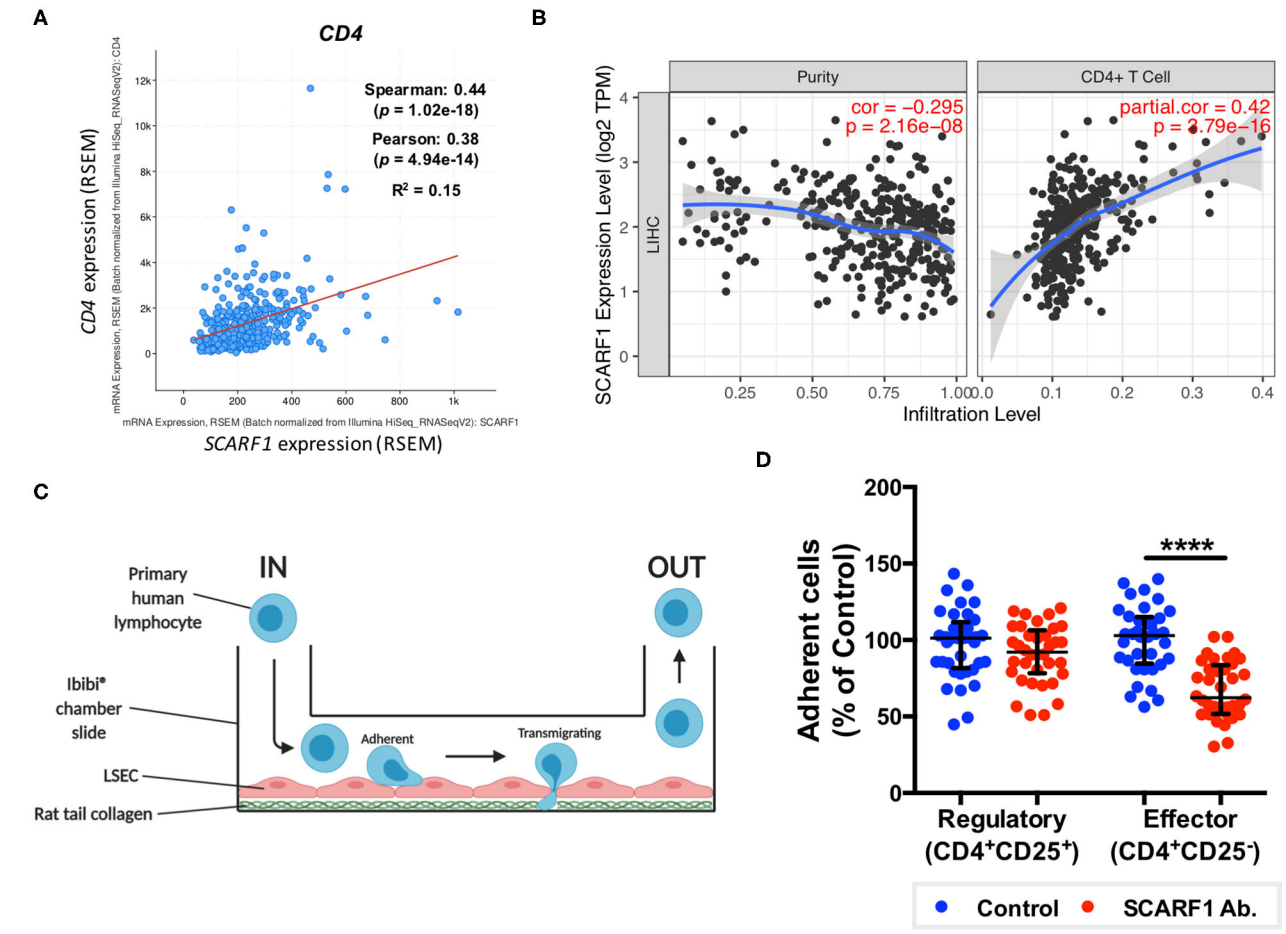
SCARF1 potentially mediates the recruitment of proinflammatory CD4^+^ T cells to HCC tumors. **(A)** Correlation of SCARF1 expression with CD4 expression in HCC tumor tissues. *n* = 358. **(B)** Correlation of SCARF1 expression with the extent of CD4^+^ T cell infiltration in HCC tumor tissues. *n* = 358. **(C)** Schematic representation of a flow-based adhesion assay with primary human lymphocytes flowed across primary human LSEC. **(D)** Quantification of percentage of adherent CD4^+^ T cell subsets [regulatory (CD4^+^CD25^+^) and effector (CD4^+^CD25^−^)] in the presence of SCARF1 blocking antibody or isotype-matched control (Control) antibody. **** indicates statistical significance as measured by a paired *T*-test, where *p* ≤ 0.001. *n* = 3 independent experiments with different LSEC and lymphocyte donors, with 12 fields of view taken from each. Data in **(B)** was generated with use of Tumor IMmune Estimation Resource (TIMER; https://cistrome.shinyapps.io/timer/; accessed 12th May 2020). Image in **(C)** created with BioRender.com.

Having previously shown that SCARF1 mediates the specific recruitment of CD4^+^ T cells to LSEC *in vitro*, under conditions of physiological flow (14), we aimed to determine whether it could play a role in the recruitment of TILs to the HCC tumor microenvironment. Firstly, and again utilizing the data available on the cBioPortal website, we correlated the expression of *SCARF1* with *CD4* expression and showed a moderate positive correlation between the two (Figure 5A). We next used a publically-available tool, Tumor IMmune Estimation Resource (TIMER; https://cistrome.shinyapps.io/timer/) to correlate *SCARF1* expression with the level of CD4^+^ T cell infiltration of HCC tumors. Using TIMER, we confirmed that *SCARF1* expression is absent from tumor cells, as indicated by a negative “purity” correlation (−0.295; Figure 5B, left panel), and demonstrated a moderate positive correlation with CD4^+^ T cell infiltration (purity-corrected partial Spearman's rho value = 0.420, *p* = 3.79e^−16^; Figure 5B, right panel). The balance of immune subsets within the tumor microenvironment plays a critical role in tumor development and progression, with an immunosuppressive microenvironment promoting immune escape and a poor prognosis (46). To assess if SCARF1 could functionally contribute to the balance of immune effector vs. immunosuppressive subsets within the tumor microenvironment, we studied its role in CD4^+^ T cell subset recruitment. Using flow-based adhesion assays under conditions of physiological shear stress with primary human LSEC and purified populations of primary human CD4^+^ T cells (Figure 5C), we showed that antibody blockade of SCARF1 on LSEC had a significant (~40%; *p* ≤ 0.001) inhibitory effect on the adhesion of proinflammatory (CD4^+^CD25^−^) T cells (effectors), but a negligible (~10%) effect on regulatory (CD4^+^CD25^+^) T cells (T_regs_) (Figure 5D).

## Discussion

Hepatocellular carcinoma (HCC) predominantly manifests on a background of cirrhosis and, consequently, in conjunction with the global rise in chronic liver diseases, incidence of HCC is also set to rise. Novel medical therapies are urgently required as patients often present to clinic with advanced tumors without curative options (47). Recently, immunotherapies have provided a number of very promising prospects in the treatment of a wide range of cancers; in particular, checkpoint inhibitors and chimeric antigen receptor (CAR)-T cell therapy have received significant attention. Checkpoint inhibitors aim to release the “brake” from the immune system, thus allowing a robust anti-tumoral host immune response (48, 49) and CAR-T cells are genetically-engineered T cells specifically designed to recognize tumor antigen-expressing cells and subsequently kill them (50). However, in solid organ tumors, both these approaches are reliant on leukocyte trafficking to the tumor and this remains a significantly under-studied aspect of cancer immunotherapy (51). In the liver, leukocyte trafficking occurs within the microvasculature, known as the hepatic sinusoids. The low shear environment leads to a unique adhesion cascade with the lack of selectin-mediated recruitment and a number of atypical adhesion receptors involved in immune cell recruitment to liver sinusoidal endothelial cells (LSEC). Our previous studies have explored immune cell recruitment in the context of chronic inflammatory liver diseases, with a particular focus on LSEC-expressed scavenger receptors; however, we have also identified members of the scavenger receptor family which are expressed *in vivo* on the endothelium of human HCC, thus suggesting that they may also contribute to immune cell recruitment to the tumor microenvironment (13, 14).

Scavenger receptors represent a major subset of innate pattern recognition receptors able to bind a number of cancer-relevant ligands, such as heat shock proteins (HSPs) (52, 53) and bacterial lipopolysaccharide (LPS) (54–56), and are known to be involved in the pathophysiology of a range of cancers, including HCC (19). Interestingly, the expression of a number of scavenger receptors is often associated with poor prognosis and less favorable clinicopathological features in HCC. For example, increased expression of CD68 and CD163, which is indicative of increased numbers of anti-inflammatory macrophages, is associated with poor overall and disease-free survival (57, 58). In addition, upregulation of the CXCL16-CXCR6 axis was associated with increased invasiveness and recurrence and, as a consequence, was also associated with poorer survival in HCC (59). In contrast to this, we show that higher intratumoral expression of SCARF1 in HCC was associated with less advanced and less aggressive cancers (Figure 2). In addition, from a prognostic perspective, higher SCARF1 expression in HCC tumors was highly indicative of better overall, disease-free and progression-free survival (Figure 3).

Consistent with our previous findings in normal and chronically diseased liver tissues (14), SCARF1 in HCC tumor tissues exhibited a highly sinusoidal expression pattern (Figure 1C) and correlation data from the TGCA dataset further corroborated its largely endothelial expression (Figure 4A). A number of the top 25 genes commonly regulated in conjunction with *SCARF1* within HCC tumor tissues were endothelial-specific (Table S1) and all demonstrated a strong positive correlation with levels of *SCARF1* expression (Figure S2). We were able to confirm protein expression of SCARF1 in tumoral sinusoidal endothelial cells through dual immunofluorescence staining with the common endothelial marker CD31 (Figure 4B) and subsequent co-localization of the two proteins (Figure 4C). Having previously shown that SCARF1 mediates the specific recruitment of CD4^+^ T cells to LSEC in inflammatory conditions (14), we next explored whether it could play a role in the recruitment of TILs to the HCC tumor microenvironment. We found that *SCARF1* expression showed a positive correlation with both *CD4* expression (Figure 5A) and the level of CD4^+^ T cell infiltration of HCC tumors (Figure 5B, right panel). Given that a loss of *SCARF1* expression was associated with more advanced and aggressive HCC tumors and that increased expression was highly prognostic of better survival, we hypothesized that SCARF1 could potentially be playing a beneficial role in the pathophysiology of HCC by shaping the immune infiltration of the tumor microenvironment (60). We used primary human LSEC in flow-based adhesion assays with CD4^+^ T cells subsets and showed that antibody blockade of SCARF1 could indeed significantly inhibit the adhesion of proinflammatory (CD4^+^CD25^−^) T cells (effectors), but had little effect the adhesion of regulatory (CD4^+^CD25^+^) T cells (T_regs_). This is in direct contrast to our previous work on another endothelial-expressed scavenger receptor, Stabilin-1, which is also present in HCC tumors, but plays a role in the specific recruitment of anti-inflammatory regulatory (CD4^+^CD25^+^) T cells (13).

These findings highlight the potential role of SCARF1 expression as a prognostic biomarker in HCC. Down regulation of *SCARF1* was associated with a poorer outcome and interestingly this may be a relevant to other tumors as we showed that tumors of other gastrointestinal cancers, such as esophageal, gastric and colonic cancers, also significantly down-regulated SCARF1 expression (Figure S1). In addition to high *SCARF1* expression correlating with a good outcome, our functional assays also suggest that SCARF1 may have an active anti-tumoral role for by promoting the recruitment of effector CD4^+^ T cells rather than tumor promoting regulatory T cells (T_regs_). This is particularly pertinent as previous studies have specifically shown that an increased prevalence of T_regs_ is an independent prognostic factor in HCC; therefore, shifting this balance could have a significant impact on patient outcome (61). In contrast to other GI malignancies, we found that pancreatic adenocarcinoma tumor tissues exhibited comparable SCARF1 expression to non-tumorous tissues (Figure S1). This could be due to the fact that pancreatic tumors are inherently and notoriously immunogenically “cold” tumors, due to a combination of low neoantigenic burden, heterogeneous dense stroma and an immunosuppressive tumor microenvironment (62). Therefore, an active downregulation of SCARF1 expression, in order to provide more favorable tumorigenic conditions, may not play a role in the pathogenesis of this tumor.

**Figure 6:**
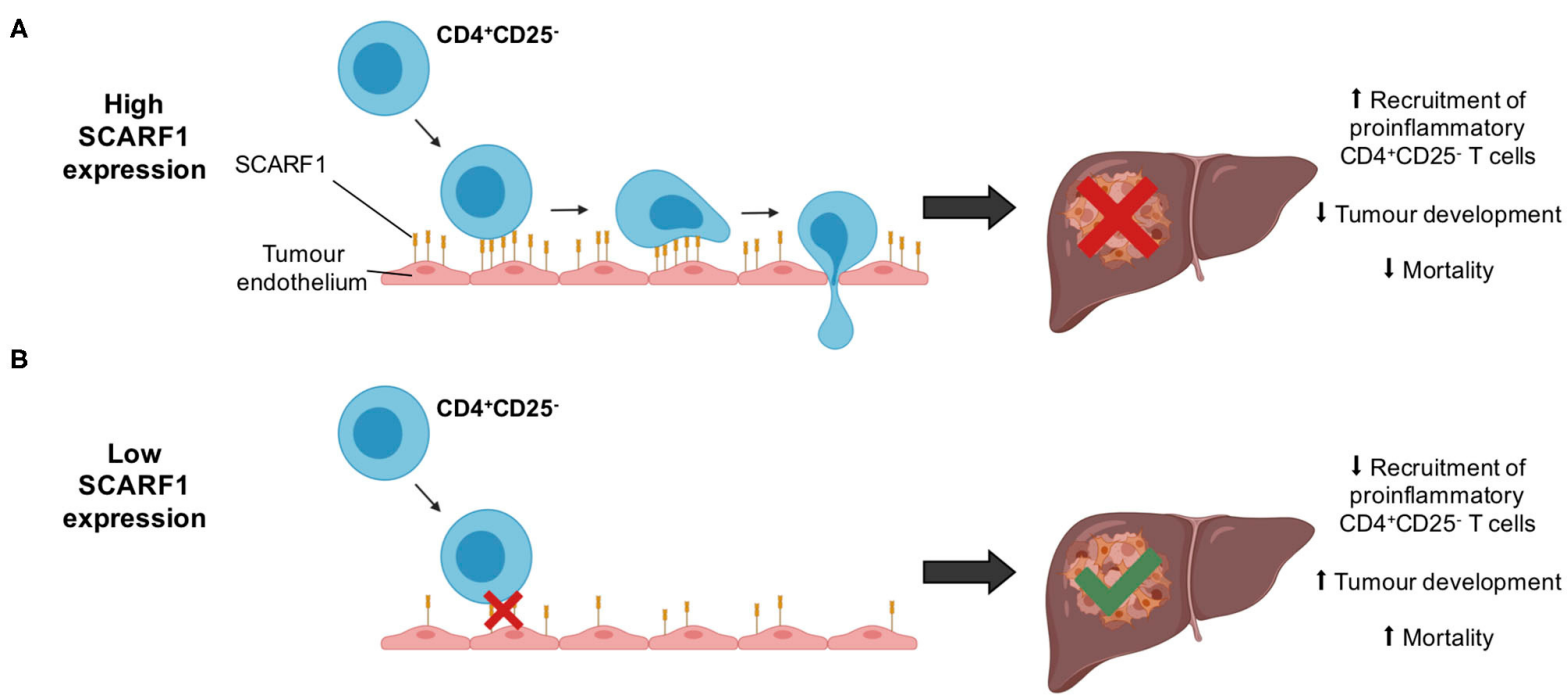
Schematic representation of potential mechanism of action of SCARF1 in HCC tumors. **(A)** In the presence of high expression of SCARF1 in tumor endothelial cells, proinflammatory CD4^+^CD25^−^ T cells are recruited to HCC tumors, resulting in decreased tumor development and, ultimately, decreased mortality. **(B)** In low expression of SCARF1 in tumor endothelial cells, recruitment of CD4^+^CD25^−^ T cells lower, resulting in increased tumor development and mortality. Image created with BioRender.com.

Whilst immunotherapy has shown exciting results in subgroups of HCC patients, there is limited stratification to support the selection of these subgroups. The correlation of SCARF1 with CD4^+^ T cell tumor infiltration and its role in recruitment may help in the identification of patients who will respond to immunotherapies. However, further *in vivo* work is now required with SCARF1 knockout models to confirm the extent of its contribution to the HCC immune microenvironment. In addition, the identity of the receptor for SCARF1 present on CD4^+^CD25^−^ lymphocytes remains unknown, thus further studies are also required to identify its ligand. Furthermore, SCARF1 is primarily a scavenger receptor, and so the impact of its presence with regards to its scavenging function also needs to be considered in future studies. SCARF1 has been shown to bind a range of endogenous ligands, such as oxidized lipoproteins (21), heat shock proteins (23–25) and apoptotic cells (26, 27), and regulate LPS responses (35); therefore, all these functions could also potentially influence the tumor microenvironment. For example, the uptake of these factors by SCARF1 could prevent neutrophil and macrophage accumulation in the tumor microenvironment, thus providing an alternative mode-of-action for the anti-tumoral action of SCARF1, as myeloid cell accumulation is often associated with poor prognosis in HCC (63, 64). Nevertheless, our data here show that SCARF1 could potentially support the recruitment of proinflammatory (CD4^+^CD25^−^) T cells to HCC tumors, leading to decreased tumoral progression and, ultimately, a better overall outcome (Figure 6). Our findings also suggest that future agonistic agents acting to increase the expression of SCARF1 within tumors could boost the numbers of tumor-infiltrating proinflammatory lymphocytes. Further experimental studies of SCARF1 could therefore lead to novel combination immunotherapeutic strategies in HCC as well as in other gastrointestinal tumors.

## Data Availability Statement

The raw data supporting the conclusions of this article will be made available by the authors, without undue reservation.

## Ethics Statement

The studies involving human participants were reviewed and approved by West Midlands–South Birmingham Research Ethics Committee. The patients/participants provided their written informed consent to participate in this study.

## Author Contributions

DP: conceptualization, formal analysis, and writing–original draft. DP, AW, and JO'R: data curation and investigation. DP and SS: funding acquisition, methodology, and writing–review and editing. All authors contributed to the article and approved the submitted version.

## Conflict of Interest

SS has received a research grant from Faron Pharmaceuticals to design a Phase I/II trial (TIETALC) of the drug “Clevergen” in patients with HCC and also reports consulting for Faron Pharmaceuticals. The remaining authors declare that the research was conducted in the absence of any commercial or financial relationships that could be construed as a potential conflict of interest.
