# Supplementary material for: Prognostic Value and Potential Immunoregulatory Role of SCARF1 in Hepatocellular Carcinoma
Source: Front Oncol. 2020 Sep 29;10:565950. doi: 10.3389/fonc.2020.565950 (PMC8336907; doi:10.3389/fonc.2020.565950)
Supplement: Supplementary file 1 [file Data_Sheet_1.DOCX]

**Figure S1 – SCARF1 expression is downregulated in gastrointestinal cancers.** Comparison of SCARF1 gene expression in non-tumoural (blue) tissues with HCC tumour tissues (red). **** indicates statistical significance where p ≤ 0.001.
