# Supplementary material for: Prognostic Value and Potential Immunoregulatory Role of SCARF1 in Hepatocellular Carcinoma
Source: Front Oncol. 2020 Sep 29;10:565950. doi: 10.3389/fonc.2020.565950 (PMC8336907; doi:10.3389/fonc.2020.565950)
Supplement: Supplementary file 2 [file Data_Sheet_2.DOCX]

**Figure S2 – Association of scavenger receptors with Overall Survival in HCC patients.** Data is displayed as hazard ratio with 95 % confidence intervals. Red plots highlight scavenger receptors where a statistically significant positive association with Overall Survival was achieved, whereas the blue plot indicates a negative association. * and ** indicate statistical significance where p ≤ 0.05 and p ≤ 0.01, respectively. n = 364. Data in this Figure was generated with use of KM Plotter (<http://kmplot.com/analysis/>).
