# Supplementary material for: Prognostic Value and Potential Immunoregulatory Role of SCARF1 in Hepatocellular Carcinoma
Source: Front Oncol. 2020 Sep 29;10:565950. doi: 10.3389/fonc.2020.565950 (PMC8336907; doi:10.3389/fonc.2020.565950)
Supplement: Supplementary file 3 [file Data_Sheet_3.DOCX]

**Figure S3 – Correlation of *SCARF1* expression with endothelial-associated genes** (A) *ADGRF5*; (B) *CD93*; (C) *FLT4*; (D) *MMRN2*; (E) *ESAM*; (F) *PEAR1*; (G) *PECAM1*; (H) *TIE1*; (I) *CLEC14A*. Correlation of *SCARF1* expression with endothelial-specific markers was performed via the cBioPortal website (<https://www.cbioportal.org/>) (accessed 25th Feb 2020). n = 358.
