# Supplementary material for: Prognostic Value and Potential Immunoregulatory Role of SCARF1 in Hepatocellular Carcinoma
Source: Front Oncol. 2020 Sep 29;10:565950. doi: 10.3389/fonc.2020.565950 (PMC8336907; doi:10.3389/fonc.2020.565950)
Supplement: Supplementary file 4 [file Table_1.DOCX]

|  | **Gene Symbol** | **Gene ID** |  |
| --- | --- | --- | --- |
| **1** | [*ADGRF5*](http://gepia.cancer-pku.cn/detail.php?gene=ADGRF5) | ENSG00000069122.18 | ***** |
| **2** | [*CD93*](http://gepia.cancer-pku.cn/detail.php?gene=CD93) | ENSG00000125810.9 | ***** |
| **3** | [*CDH5*](http://gepia.cancer-pku.cn/detail.php?gene=CDH5) | ENSG00000179776.17 |  |
| **4** | [*ERG*](http://gepia.cancer-pku.cn/detail.php?gene=ERG) | ENSG00000157554.18 |  |
| **5** | [*FLT4*](http://gepia.cancer-pku.cn/detail.php?gene=FLT4) | ENSG00000037280.15 | ***** |
| **6** | [*PCDH12*](http://gepia.cancer-pku.cn/detail.php?gene=PCDH12) | ENSG00000113555.5 |  |
| **7** | [*MMRN2*](http://gepia.cancer-pku.cn/detail.php?gene=MMRN2) | ENSG00000173269.13 | ***** |
| **8** | [*LDB2*](http://gepia.cancer-pku.cn/detail.php?gene=LDB2) | ENSG00000169744.12 |  |
| **9** | [*MYCT1*](http://gepia.cancer-pku.cn/detail.php?gene=MYCT1) | ENSG00000120279.6 |  |
| **10** | [*ARHGEF15*](http://gepia.cancer-pku.cn/detail.php?gene=ARHGEF15) | ENSG00000198844.10 |  |
| **11** | [*CYYR1*](http://gepia.cancer-pku.cn/detail.php?gene=CYYR1) | ENSG00000166265.11 |  |
| **12** | [*ESAM*](http://gepia.cancer-pku.cn/detail.php?gene=ESAM) | ENSG00000149564.11 | ***** |
| **13** | [*PEAR1*](http://gepia.cancer-pku.cn/detail.php?gene=PEAR1) | ENSG00000187800.13 | ***** |
| **14** | [*PECAM1*](http://gepia.cancer-pku.cn/detail.php?gene=PECAM1) | ENSG00000261371.5 | ***** |
| **15** | [*TIE1*](http://gepia.cancer-pku.cn/detail.php?gene=TIE1) | ENSG00000066056.13 | ***** |
| **16** | [*BCL6B*](http://gepia.cancer-pku.cn/detail.php?gene=BCL6B) | ENSG00000161940.10 |  |
| **17** | [*CLEC14A*](http://gepia.cancer-pku.cn/detail.php?gene=CLEC14A) | ENSG00000176435.6 | ***** |
| **18** | [*GIMAP8*](http://gepia.cancer-pku.cn/detail.php?gene=GIMAP8) | ENSG00000171115.3 |  |
| **19** | [*MEF2C*](http://gepia.cancer-pku.cn/detail.php?gene=MEF2C) | ENSG00000081189.13 |  |
| **20** | [*RP11-389C8.2*](http://gepia.cancer-pku.cn/detail.php?gene=RP11-389C8.2) | ENSG00000261269.1 |  |
| **21** | [*SH2D3C*](http://gepia.cancer-pku.cn/detail.php?gene=SH2D3C) | ENSG00000095370.19 |  |
| **22** | [*TCF4*](http://gepia.cancer-pku.cn/detail.php?gene=TCF4) | ENSG00000196628.13 |  |
| **23** | [*FAM198B*](http://gepia.cancer-pku.cn/detail.php?gene=FAM198B) | ENSG00000164125.15 |  |
| **24** | [*ADAMTS7*](http://gepia.cancer-pku.cn/detail.php?gene=ADAMTS7) | ENSG00000136378.14 |  |
| **25** | [*CD34*](http://gepia.cancer-pku.cn/detail.php?gene=CD34) | ENSG00000174059.16 |  |

**Table S1 – Top 25 genes regulated in conjunction with *SCARF1* in HCC tumours.** * indicates endothelial-specific genes selected for correlation analysis in Figure 4. This list was generated by use of the Gene Expression Profiling Interactive Analysis (GEPIA) website (<http://gepia.cancer-pku.cn/>).
